# Nasal exhaled nitric oxide measurements on British Asian children with confirmed Primary Ciliary Dyskinesia

**DOI:** 10.1186/2046-2530-1-S1-P9

**Published:** 2012-11-16

**Authors:** E Turner, M Balain, EF Moya, P Dawson

**Affiliations:** 1 Bradford Royal Infirmary, UK

## Introduction

The prevalence of Primary Ciliary Dyskinesia (PCD) in British Asian children in Bradford is 1 in 2265. This is the highest in the UK and more common than Cystic Fibrosis. 1) The use of Nasal Exhaled Nitric Oxide (ENO) as a screening tool for PCD was a suggested by ATS/ERS recommendations in 2005. 2) We have collected values for British Asian children with PCD in Bradford.

## Methods

26 children in Bradford have a confirmed diagnosis of PCD by Nasal Cilia brushings. 24 children are of British Asian origin. We obtained nasal ENO values for 16 children.

## Results

14/16 children with confirmed PCD had nasal ENO values <100.

## Summary

Our results in British Asian children agree with evidence suggesting Nasal ENO may be used as a screening tool for PCD. Next step, The Bradford Exhaled Nitric Oxide (BENO) Study aims to generate normal reference nasal ENO values from healthy School aged British Asian children in Bradford. If values in children with PCD are consistently lower than in healthy children, we hope to introduce nasal ENO as a screening tool for PCD for British Asian Children in Bradford.
